# Some Diseases of the Male Genital System

**Published:** 1908-01-18

**Authors:** 


### SOME DISEASES OF THE MALE GENITAL SYSTEM.

### V.—HÆMATOCELE.

This is an effusion of blood into the tunica vaginalis. It is commonly ascribed to an injury affecting the part, and the patient nearly always gives such a history. It may be taken that when a patient has received an injury to the testis sufficiently grave to give rise to a hæmatocele, the pain caused by the blow will not be readily forgotten. But in other cases the history is less reliable, the patient conjuring up an injury out of his imagination to account for his observed symptoms. It is the tendency of human nature to do this, and a history of antecedent injury as the cause of a present condition must be received with caution in all surgical cases. If this view is correct we may say that there are certain cases in which a definite injury has occurred of sufficient gravity to rupture one or more of the vessels in a previously healthy tunica vaginalis. But how are we to explain the cases in which no such history can be obtained? Either on the ground that the vessels of the tunica vaginalis are diseased and give way spontaneously in a manner analogous to that in which the cerebral vessels do in cerebral hæmorrhage, or that an injury so slight as to be unnoticed at the time has been sufficient to produce a similar result in vessels already diseased, or especially liable to damage. As an example of the latter some authorities believe that the predisposing condition is an inflammatory one, which has led to the formation of adhesions, and these contain delicate vessels which are unsupported, and thus easily ruptured. Against this view, however, it must be said that the presence of adhesions, the result of old inflammation, is not so common in the tunica vaginalis as it is in other serious membranes; and very few are found even in cases where a hydrocele has been present which has been subjected to repeated tappings, or to the injection of irritant solutions.

The condition of the tunica vaginalis in cases of hæmatocele varies according to the length of time during which the affection has persisted. In old-standing cases in which the effused blood has clotted and become organised, the wall is thick and firm, and may contain partly-formed or fully-developed fibrous tissue, or it may even be calcified. In such the cavity in the centre may be quite small, or, more rarely, there may be none at all, the whole tumour having become solid. The fluid contained in the centre varies, as does the state of the walls, according to the duration of the condition: in recent cases it more or less resembles freshly-shed blood, but in old-standing ones it is a chocolate brown colour, and thick in consistence like treacle. If the hæmorrhage has occurred into an already existing hydrocele the fluid may be quite clear, and bright red. This is a point which should be borne in mind in the differential diagnosis between hæmatocele and sarcoma of the testis. In the latter disease there is often secondarily some blood in the tunica vaginalis; and some surgeons advocate making the diagnosis certain by aspirating. If blood is drawn off which is sticky and dark brown, it may be safely assumed that a hæmatocele is the cause of the trouble. The blood withdrawn from the tunica in sarcoma cases is bright red, and resembles in consistency ordinary blood; the last point distinguishes it from hæmorrhage into a hydrocele, the fluid here being thin and watery. A hydrocele may be converted into a hæmatocele by a blow, or as the result of tapping. In spite of extreme care to avoid every obvious vessel during the insertion of the trocar, hæmorrhage sometimes ensues from accidental puncture; and in other cases the sudden withdrawal of fluid followed by the collapse of distended and weakened vessels may lead to hæmorrhage, just as hæmaturia may occur after the catheterisation of an acutely distended bladder.

The onset of a hæmatocele is most often sudden, and in many cases is, as has been said, attributed by the patient to a definite blow or injury. The swelling, which at first fluctuates, but becomes solid later, may or may not be painful. When present it is generally due to the more or less sudden distension of the tunica vaginalis with fluid for which it was not prepared. Translucency, of course, is absent, and aspiration results in the withdrawal of blood, altered in character to a varying degree. The swelling which characterises a hæmatocele is of the same shape as a hydrocele; but whereas a hydrocele feels uniform and elastic a hæmatocele feels lumpy, being hard in some places and soft in others; moreover, there may be discoloration of the skin of the scrotum owing to effusion of blood into the subcutaneous tissues. In quite a small proportion of cases the onset is insidious, and these are the difficult ones to diagnose, because they may easily be mistaken for disease of the body of the testis itself.

Suppuration is not uncommon in hæmatocele. It is generally the result of irritation or further injury, which predisposes to a hæmic infection. The swelling then shows all the characteristic phenomena of acute inflammation and tends to point at one spot.

A recent hæmatocele which is definitely traumatic in origin should be treated with complete rest, suspension of the scrotum, and the application of an ice-bag to the part. If the swelling is not absorbed, a small incision should be made and the blood evacuated. It is not wise to tap a hæmatocele: nothing is more easily infected with pyogenic micro-organisms than a collection of effused blood; and in scrotal cases the risk is doubly great. In old-standing hæmatoceles with thick walls it is hopeless to expect a cure by merely incising them and turning out the contained blood clot. The cavity will merely refill. Nothing short of complete excision of the whole parietal layer of the tunica vaginalis is of any avail. Great care must be taken not to injure the vas; in the altered condition of the part, it may be difficult to recognise. If the wall is calcareous, it is probably best to perform castration, since the pressure of the swelling on the testis has probably been sufficient to render that organ functionless.

